# Heteropolyacid supported on ionic liquid decorated hierarchical faujasite zeolite as an efficient catalyst for glycerol acetalization to solketal

**DOI:** 10.1038/s41598-023-42956-8

**Published:** 2023-09-21

**Authors:** Samahe Sadjadi, Sara Tarighi, Motahareh Delangiz, Majid Heravi

**Affiliations:** 1 Gas Conversion Department, Faculty of Petrochemicals, Iran Polymer and Petrochemical Institute, P.O. Box 14975-112, Tehran, Iran; 2 Department of Chemistry, School of Physic and Chemistry, Alzahra University, P.O. Box 1993891176, Vanak, Tehran, Iran

**Keywords:** Chemistry, Catalysis, Heterogeneous catalysis

## Abstract

To handle huge amount of glycerol produced in biodiesel industry, glycerol is transformed to value-added products. In this regard, glycerol acetalization to solketal is industrially attractive. As in this process various by-products can be formed, designing highly selective catalysts is of great importance. In this line, we wish to report a novel catalyst that benefits from strong acidity, high specific surface area and thermal stability, which can selectively form solketal in glycerol acetalization. To prepare the catalyst, hierarchical zeolite was prepared via a novel method, in which partially dealuminated NaY was treated with PluronicF-127 and then reacted with NH_4_NO_3_ to furnish the H-form zeolite. Hierarchical faujasite was then achieved through calcination and template removal. Subsequently, it was functionalized with ionic liquid and used for the immobilization of heteropolyacid. The results indicated the importance of the mesoprosity of zeolite and the presense of ionic liquid functionality for achiveing high solketal yield. Moreover, among three investigated heteropolyacids, phosphomolybdic acid exhibited the highest catalytic activity. In fact, using 10 wt% catalyst at 55 °C and glycerol to acetone molar ratio of 1:20, solketal with yield of 98% was furnished under solvent-less condition. Besides, the catalyst was recyclable with low leaching of heteropolyacid.

## Introduction

**Figure 1:**
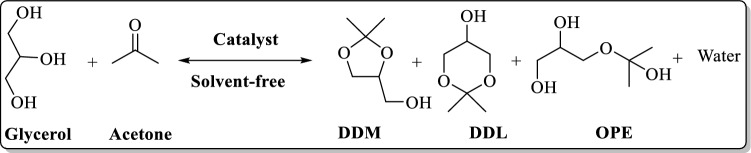
Catalytic acetalization of glycerol.

Environmental concerns as well as depletion of fossil fuels led to the growing utilization of biodiesel. Huge production of biodiesel consists of some challenges, such as handling of glycerol that is the main by-product of biodiesel production. As a solution to this problem, glycerol can be transformed to value-added products, such as fuel additives^1–4^. In this regard, synthesis of 2, 2-dimethyl-1, 3-dioxolane-4-methanol, mostly known as solketal (DDM) has received immense attraction^5–7^. DDM that is applied as a fuel-additive can be achieved through acetalization of glycerol. The main challenging issues in this chemical transformation are formation of several (by)products, such as 2,2-dimethyl-1,3-dioxane-5-ol (DDL) and 3-(2-oxida- nylpropan-2-yloxy)propane-1,2-diol (OPE)^8–10^, Fig. 1, and relatively long reaction time and high temperatures^9,11^. To address these issues, various catalysts have been reported^12–15^. In this regard, heterogeneous catalysts that can selectively catalyze the reaction under mild reaction condition to give DDM are highly desirable.

One of the promising catalysts for glycerol acetalization is heteropolyacids, HPAs. These non-corrosive and non-toxic inorganic acids^16^ are extensively applied in the catalysis^17^. In fact, HPAs are considered as green alternatives for conventional acidic catalysts. Notably, the possibility of designing wide range of HPAs as well as the redox potential of these compounds render them potent catalysts for promoting diverse range of chemical, electrochemical and photochemical transformations^18–21^. HPAs can be classified into various sub-classes, among which Keggin is one of the most well-known structures. The main drawbacks of HPAs is their high solubility in protic and aprotic solvents, which renders it a homogeneous catalyst. To circumvent this problem, HPA is mostly immobilized on a support.

In this regard, aluminosilicate zeolites have been extensively applied for the development of catalysts^22–24^ with utility for chemical and petrochemical processes due to their favourable features, such as porosity, thermal stability, acidity and tune-ability^25^. However, conventional zeolites that are microporous, restrict mass transfer and accessibility to the active sites and consequently negatively affect the catalytic performance of the catalysts^26–28^. To circumvent this deficiency, various approaches have been suggested, among which synthesis of hierarchical zeolites that possess both micropores and mesopores has received immense attention^29–34^. Hierarchical zeolites can be prepared through top-down (post-synthesis) and bottom-up synthetic approaches. In top-down approach, the framework cations are partially extracted to form defects of meso/macropore dimensions in zeolite crystals. The bottom-up approach is fulfilled through integration of template, mostly surfactants, in the growing crystals, followed by template removal. The hierarchical zeolites with an interpenetrating micro/mesoporous system, are highly promising as acid catalysts. Notably, the nature of the used surfactants can affect the features of the resulting hierarchical zeolites^35^. In fact, surfactants with the capability to form large micelles or micelle aggregates with small curvatures, such as triblock polymer of P123, triblock polymer of F127 are highly desirable to form mesopores in the structure of zeolites^36^.

It is worth mentioning that the properties of the supporting materials can also be modified through chemical functionalization^37,38^. One of the mostly studied functional groups that has been widely utilized for the decoration of supporting materials is ionic liquid, IL^39,40^. As ILs are composed of electrically charged anions and cations, they can electrostatically interact with catalytic species and stabilize them on the support^41^.

In the continuation of our efforts on designing of task-specific zeolites^15,42,43^ and catalysts^44,45^, in this article we wish to report a novel and facile route for the controlled synthesis of ultra-stable hierarchical zeolite Y with large mesopores (HR/Y-F127) by using nonionic amphiphilic copolymer PluronicF-127 as template. The as-prepared HR/Y-F127 was then covalently modified with IL and then used as a support for the stabilization of phosphomolybdic acid (HPMo) to furnish HR/Y-F127-IL-HPMo. The catalyst was then characterized and applied for promoting production of DDM from acetalization of glycerol under mild condition. A precise study on the effects of the reaction temperature, catalyst loading and nature of HPA has been carried out to optimize the reaction conditions. Moreover, the roles of IL and the hierarchical structure of the zeolite in the activity of the catalyst as well as recyclability of the catalyst and the reaction mechanism have been studied.

## Result and discussion

### Characterization of HR/Y-F127-IL-HPMo

**Figure 2:**
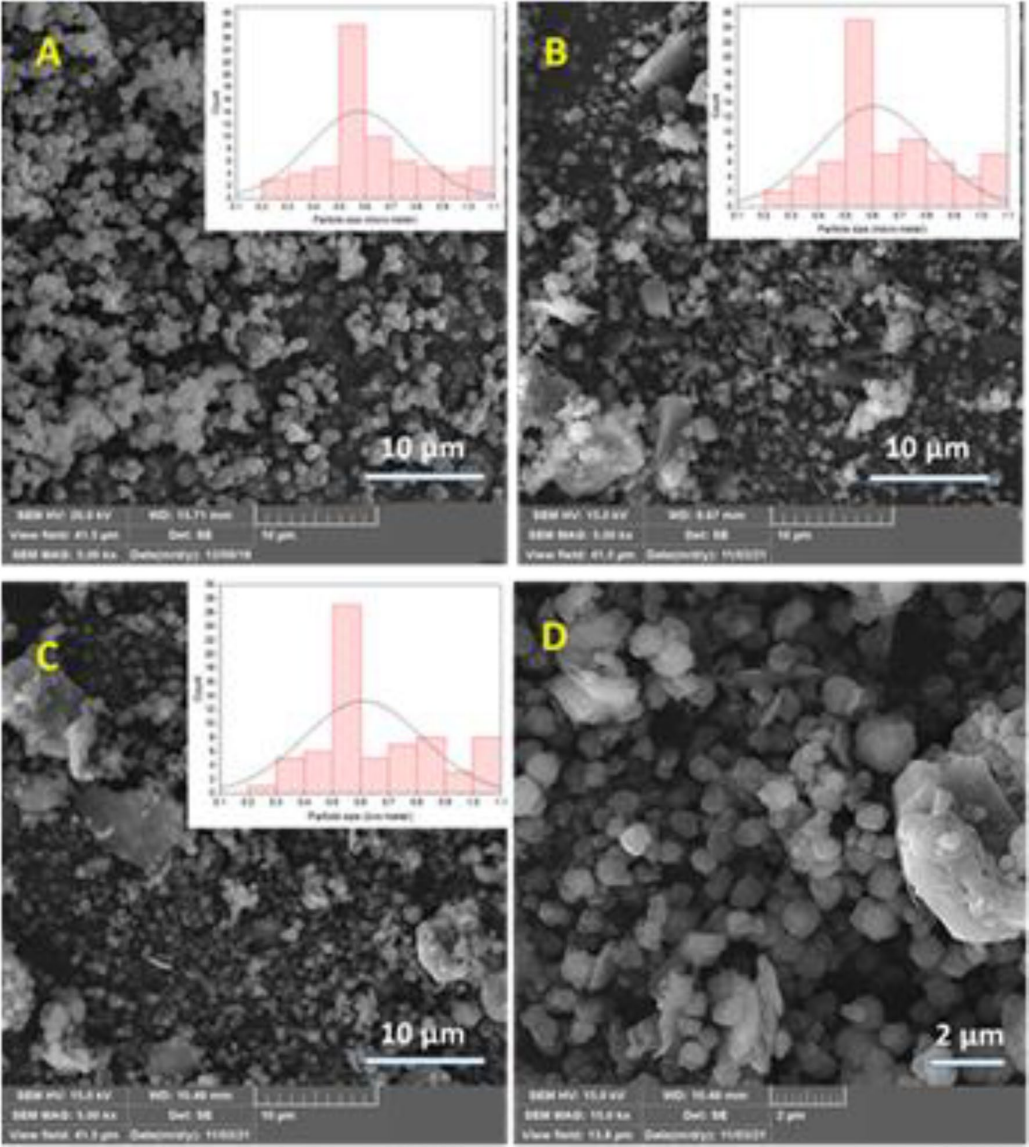
SEM images of (**A**) NaY, (**B**) HR/Y-F127, (**C**, **D**) HR/Y-F127-IL-HPMo.

Using SEM, the morphological studies have been accomplished. In this regard, SEM images of NaY, HR/Y-F127 and HR/Y-F127-IL-HPMo have been recorded and compared. As displayed in Fig. 2 A, the as-synthesized NaY exhibits cubic morphology. This observation is in a good accordance with the literature^46^ and approves successful formation of NaY zeolite framework. In the SEM image of HR/Y-F127 (Fig. 2B), small cubic particles with sizes of 0.5–1 μm along with small aggregates are detectable. In fact, the similarity of the morphology of NaY and HR/Y-F127 confirms the stability of zeolite structure upon modification. As shown in Fig. 2C, D, the morphology of HR/Y-F127-IL-HPMo is very similar to that of HR/Y-F127, implying that modification of HR/Y-F127 with IL and HPMo impregnation did not significantly affect the morphology.

The results of EDS and elemental mapping of HR/Y-F127-IL-HPMo is represented in Fig. 1S. As illustrated in Figure S1 (A), N, C, O, Al, Si, Mo, P, Cl and Na are present in the structure of the as-prepared HR/Y-F127-IL-HPMo. Among the observed atoms, P, O and Mo can be assigned to HPMo, while C, N, O and Cl can confirm conjugation of the IL moiety. Noteworthy, Si, O, Na and Al atoms can be ascribed to HR/Y-F127. Elemental mapping of HR/Y-F127-IL-HPMo, Figure S1 (B), shows high dispersion of Cl, C and N atoms, implying that IL moiety has been uniformly grafted on HR/Y-F127. Similarly, homogeneous dispersion of Mo and P atoms confirms that HPMo has been well-distributed on HR/Y-F127-IL.

**Figure 3:**
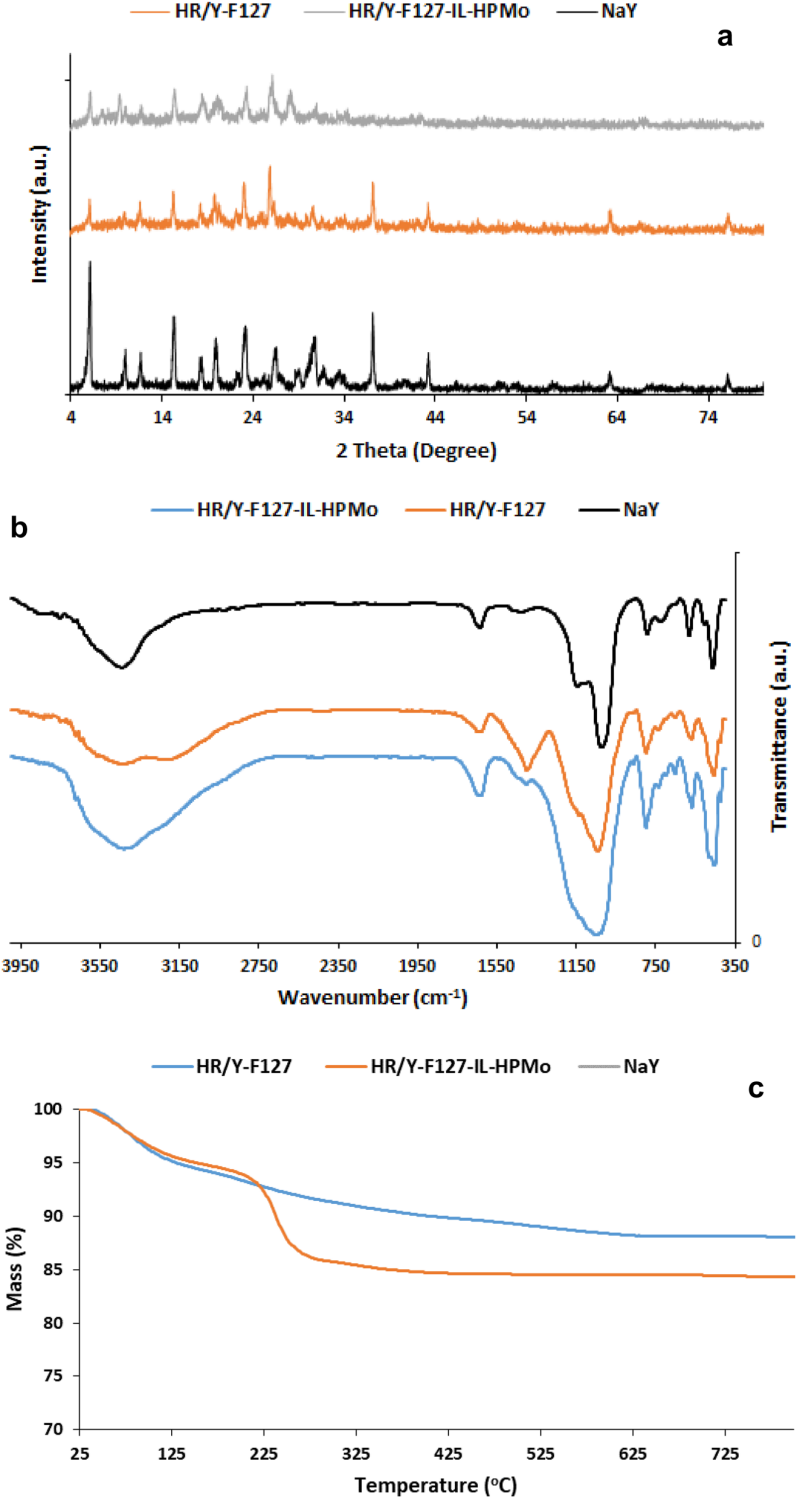
(**a**) XRD patterns of NaY, HR/Y-F127 and HR/Y-F127-IL-HPMo; (**b**) FTIR spectra of NaY, HR/Y-F127 and HR/Y-F127-IL-HPMo; (**c**) TG curves of HR/Y-F127 and HR/Y-F127-IL-HPMo.

XRD patterns of NaY, HR/Y-F127 and HR/Y-F127-IL-HPMo are presented in Fig. 3a. The XRD pattern of HR/Y-F127 showed all of the characteristic peaks of NaY zeolite^15^, indicating the formation of NaY phase and its stability in the course of introduction of mesopores. However, the XRD pattern of HR/Y-F127 showed peaks of lower intensity, which is due to the steam calcination of the hierarchical zeolite detracting the crystallinity of the framework. This observation is in good accordance with the previous reports^47^. Notably, the slight shift observed in the 2θ values is attributed to the immobilization of the catalytic species on the surface of zeolite. It is worth noting that no peak was detected for HPMo, which is indicative of its high dispersion on HR/Y-F127-IL^48,49^.

In Fig. 3b, FTIR spectra of NaY, HR/Y-F127 and HR/Y-F127-IL-HPMo are compared. According to the literature^50^, the characteristic bands of NaY zeolite are the band at 458 cm^−1^ that is representative of the structure-insensitive T–O bending modes to the TO_4_ tetrahedra (T = Si, Al), the band at 582 cm^−1^, which is due to the double ring external linkage peak associated with the faujasite framework, the bands at 715 and 1020 cm^−1^ that are attributed to the symmetric and asymmetric stretching vibrations of interior TO_4_ units, the band at 1633 cm^−1^ that corresponds to the scissor vibration resulting from the proton vibration in the H_2_O molecule, the bands at 788 and 1137 cm^−1^, which are indicative of the symmetric and asymmetric stretching vibrations of outer TO_4_ unit. As shown, both HR/Y-F127 and HR/Y-F127-IL-HPMo exhibit the characteristic bands of faujasite zeolite, indicating the stability of the zeolite in the course of mesopore introduction, chemical modification and HPMo impregnation. Notably, in the FTIR spectrum of HR/Y-F127-IL-HPMo, the characteristic bands of HPMo^51^, i.e. the bands at 1060 ν(P-Oa), 968 ν(Mo-Od), 876 ν(Mo-Ob-Mo), 789 cm^−1^ ν(Mo-Oc-Mo) as well as the characteristic band of IL, i.e. the band at 1633 cm^−1^ (C-N functionality) overlapped with the absorbance bands of HR/Y-F127-IL.

Thermal stability of HR/Y-F127 and HR/Y-F127-IL-HPMo were studied by recording their TG curves in the range of 25–800 °C. As illustrated in Fig. 3c, HR/Y-F127 showed high thermal stability and its TG curve exhibits only a weight loss due to the loss of structural water at ~ 100 °C. The TG curve of HR/Y-F127-IL-HPMo is distinguished from that of HR/Y-F127 and showed a weight loss at 240 °C (~ 10 wt%) that is attributed to the degradation of the IL moiety. Regarding to HPMo, loss of water molecules at temperatures of approximately 70, 90, 450 °C is expected, which is overlapping with weight losses of zeolite and IL. According to the literature, HPMo converts to a mixture of oxide of P and Mo at about 590 °C^52^.

Using NH_3_-TPD, the acidity characteristics of NaY, HR/Y-F127 and HR/Y-F127-IL-HPMo was studied and compared, Figure S2. The strength of different acidic sites was categorized by the temperature of desorbed NH_3_ peaks. In this basis, three different regions of weak, medium and strong acid sites are defined at 100–330, 300–450 and > 450 °C, respectively. As listed in Table 1, NaY zeolite exhibited one desorption peak at 188 °C which is categorized as weak acidic site. HR/Y-F127 has stronger acidic features showing two peaks at 290 °C and 415 °C that are indicative of weak and medium acid sites, respectively. The higher acidic characteristics of HR/Y-F127 rather than the parent NaY can be assigned to the dealumination process in which extra-framework Al species with acidity characteristics are produced causing stronger acidity in the zeolite framework. In the case of HR/Y-F127-IL-HPMo, two desorption peaks of ammonia were observed at significantly higher temperatures, 427 °C (medium acid sites) and 800 °C (strong acid sites), indicating that immobilization of HPMo, which is a catalytic species with strong acidity could affect the acidity of HR/Y-F127 and HR/Y-F127-IL-HPMo showing stronger acidity compared to HR/Y-F127. As tabulated, total acidity of HR/Y-F127-IL-HPMo is 4507.9 µmol/g.cat that is significantly (1811.6 µmol/g.cat) higher than that of HR/Y-F127 (2696.3 µmol/g.cat). As mentioned, higher acidity of HR/Y-F127-IL-HPMo can be attributed to the impregnated HPMo. In fact, the acidic characteristic of HPMo can contribute to the acidity of the final catalyst. On the other hand, the electrostatic interactions of HPMo and HR/Y-F127-IL that resulted in the protonation of –OH functionality of the catalyst as well as the presence of IL moiety on the structure of HR/Y-F127-IL-HPMo can enhance the acidity of the catalyst.

**Table 1:** NH_3_-TPD results for HR/Y-F127 and HR/Y-F127-IL-HPMo.

Catalyst	Weak (100–300 °C)		Medium (300–450 °C)		Strong (> 450 °C)		Total acidity (µmol/g cat)
	Peak^a^	Acidity^b^	Peak^a^	Acidity^b^	Peak^a^	Acidity^b^	
NaY	188	1304	–	–	–	–	1304
HR/Y-F127	290	1299	415	1397.3	–	–	2696.3
HR/Y-F127-IL-HPMo	–	–	427	2757.3	800	1750.6	4507.9

^a^NH_3_ peak position.

^b^Acidity amounts (NH_3_/Cat, µmol/g).

### Effect of the porosity of the support on the catalytic activity

As mentioned in the introduction section, using PluronicF-127 as a template, a novel strategy has been developed for providing mesopores in NaY zeolite. To confirm this issue, the textural properties, i.e. specific surface area, V_p_, V_meso_, V_micro_ and D_p_ of HR/Y-F127 were measured and compared with those of NaY. These values were also investigated for HR/Y-F127-IL-HPMo to elucidate the effects of functionalization and HPMo immobilization on the textural properties. As tabulated in Table 2, the specific surface area of HR/Y-F127 (324 m^2^/g) is lower than that of NaY (910 m^2^/g). Upon introduction of IL and HPMo, this value further decreased to 235 m^2^/g. This issue approves grafting of IL and stabilization of HPMo on the surface of HR/Y-F127. Comparison of V_meso_ for the three samples showed that this value for HR/Y-F127 (0.12 cm^3^/g) is significantly higher than NaY (0.03 cm^3^/g), indicating the successful formation of some mesopores along with micropores in the structure of the zeolite. In the case of HR/Y-F127-IL-HPMo, this value decreased to 0.09 cm^3^/g. This decrement can be attributed to the penetration of IL and encapsulation of HPMo in the mesopores of HR/Y-F127-IL. Similarly, decrement of V_micro_ value in HR/Y-F127 (0.25 cm^3^/g) compared to that of NaY (0.34 cm^3^/g) can establish transformation of some micropores to mesopores in HR/Y-F127. V_micro_ value for HR/Y-F127-IL-HPMo is lower than that of HR/Y-F127, which is a proof for coverage of pores with IL and HPMo. Notably, in the treated samples, i.e. HR/Y-F127 and HR/Y-F127-IL-HPMo there are both micropores and mesopores. In fact, the presence of micropores guaranteed the hierarchical structure of the zeolite^53^. Regarding D_P_, it was also discerned that modification of NaY zeolite through post-synthesis approach can considerably affect D_P_. In fact, this value in HR/Y-F127 is remarkably larger than that of NaY, demonstrating the enlargement of the pores and formation of some mesopores in the zeolite. The N_2_-adsorption–desorption isotherms of both HR/Y-F127 and HR/Y-F127-IL-HPMo are of type I (Figure S3).

**Table 2:** Textural properties of NaY, HR/Y-F127 and HR/Y-F127-IL-HPMo.

Catalyst	S_BET_ (m^2^/g)	V_P_ (cm^3^/g)	V_meso_ (cm^3^/g)	V_micro_ (cm^3^/g)	D_P_ (nm)
NaY	910	0.37	0.03	0.34	< 2.0
HR/Y-F127	324	0.37	0.12	0.25	3.92
HR/Y-F127-IL-HPMo	235	0.28	0.09	0.19	3.92

All of these results confirmed the efficiency of the present methodology for the formation of mesopores in the zeolite. To elucidate the effect of mesoporosity on the catalytic activity and DDM selectivity, the catalytic performance of NaY and HR/Y-F127 for glycerol acetalization was studied and compared with that of HR/Y-F127-IL-HPMo, Table 3. For this purpose, glycerol acetalization was carried out by using 10 wt% of catalyst under solvent-free condition at 55 °C and glycerol to acetone molar ratio of 1:20. As tabulated, NaY zeolite showed moderate catalytic activity and low selectivity towards DDM. In the case of HR/Y-F127, both conversion and DDM yield improved significantly, indicating the role of mesoporosity in the catalysis. According to the literature^54^, this issue can be assigned to the improvement of mass transfer in HR/Y-F127 that benefits from larger pores. Notably, the activity and DDM selectivity of HR/Y-F127-IL-HPMo are superior to those of HR/Y-F127. This issue can be ascribed to the roles of HPMo and IL in the catalysis. In more detail, as NH_3_-TPD confirmed, modification of HR/Y-F127 with IL and impregnation of HPMo can significantly increase the acidic strength of the catalyst, which can result in higher activity and selectivity of the catalyst.

**Table 3:** Comparison of the catalytic activity and DDM selectivity of control catalysts for glycerol acetalization with those of HR/Y-F127-IL-HPMo.

Catalyst	Conversion (%)	DDM yield (%)
NaY	44	20
HR/Y-F127	90	70
HR/Y-F127-IL-HPMo	100	98

### Effect of HPA nature

**Figure 4:**
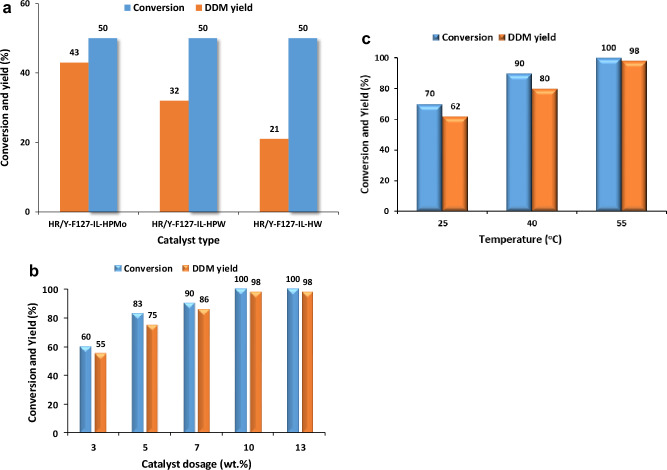
(**a**) The conversion and DDM yield of glycerol acetalization in the presence of HR/Y-F127-IL impregnated with three different HPAs. Reaction condition: glycerol to acetone molar ratio of 1:20, catalyst loading of 10 wt% at 55 °C under solvent-less condition; (**b**) the effect of HR/Y-F127-IL-HPMo dosage on the conversion and the DDM yield in acetalization of glycerol. Reaction condition: solvent-less condition, molar ration of glycerol to acetone: 1:20, at 55 °C; (**c**) the effect of reaction temperature on the conversion and the DDM yield in acetalization of glycerol. Reaction condition: solvent-less condition, molar ration of glycerol to acetone: 1:20, HR/Y-F127-IL-HPMo dosage of 10 wt%.

One of the effective factors on the catalytic activity of the HPA-based catalysts is the nature of the used HPA. To study the effect of this parameter, three Keggin type HPAs, including HPMo, HPW and HW, have been impregnated on HR/Y-F127-IL to furnish HR/Y-F127-IL-HPW, HR/Y-F127-IL-HPMo and HR/Y-F127-IL-HW catalysts. Subsequently, the catalytic activity of the three as-prepared catalysts has been investigated for the glycerol acetalization. For the sake of comparison, all experiments have been carried out under similar conditions (reaction temperature = 55 °C, glycerol to acetone molar ratio of 1:20, catalyst loading of 10 wt% under solvent-less condition). Measuring the DDM yield after 60 min, Fig. 4a, demonstrated that altering the nature of HPA did not affect the reaction conversion. However, DDM yield was slightly affected by the type of the impregnated HPA. As illustrated in Fig. 4a, DDM yield increased in the order of HR/Y-F127-IL-HPMo > HR/Y-F127-IL-HPW > HR/Y-F127-IL-HW. Considering the importance of DDM, HPMo was selected as the most efficient HPA. The origin of different catalytic activities of the used Keggin type HPAs can be ascribed to several factors. According to the literature^55^, the acidity of the Keggin type HPAs followed the order of HPW > HW > HPMo. Hence, it is expected that HPW, which possesses the highest acidity showed the best glycerol conversion and DDM selectivity. However, apart from acidity of HPAs, other factors can play role in the catalytic activity and selectivity of HPA-based catalysts. More accurately, for the supported Keggin type HPAs, the textural property of the support, such as its specific surface area and porosity can affect the loading of different Keggin type HPAs^56^, and consequently their catalytic performance.

### Effect of reaction variables

#### Effect of dosage of HR/Y-F127-IL-HPMo

Our initial studies affirmed that the presence of the catalyst is essential for achieving high conversion and DDM yield in acetalization of glycerol. Therefore, it is postulated that the dosage of HR/Y-F127-IL-HPMo can be an influential factor on this reaction. To elucidate this assumption, the effect of this parameter on the glycerol acetalization was appraised by repeating the reaction at 55 °C and glycerol to acetone molar ratio of 1:20 under solvent-less condition in the presence of different dosages of HR/Y-F127-IL-HPMo. The results, depicted in Fig. 4b, indicate the importance of the HR/Y-F127-IL-HPMo loading value on both reaction conversion and DDM yield. More precisely, increase of HR/Y-F127-IL-HPMo dosage from 3 to 10 wt% led to the increase of the reaction conversion from 60 to 100% and DDM yield from 55 to 98%. Noteworthy, 10 wt% HR/Y-F127-IL-HPMo was the optimum dosage of the catalyst and further increment of this value altered neither conversion nor DDM yield.

#### Effect of reaction temperature

Reaction temperature is one of the reaction variable that can potentially affect the reaction rate. To appraise the effect of this parameter on the glycerol acetalization and optimize it, this reaction was conducted using 10 wt% HR/Y-F127-IL-HPMo under solvent-less condition and glycerol to acetone molar ratio of 1:20 at three different temperatures (25, 40 and 55 °C) and the reaction conversion and DDM yield were estimated for each reaction. Comparison of the outcomes, Fig. 4c, implied the importance of this parameter. In more detail, glycerol acetalization at low temperatures (25 and 40 °C) resulted in low conversions and DDM yields, while increase of the reaction temperature to 55 °C led to the increase of the aforementioned values.

### Role of IL in the catalysis

Considering high solubility of HPA, its stabilization on conventional supports using classic stabilization approaches is not very effective and the stabilized HPA can easily leach from the support. This issue leads to low recyclability of the catalyst. Two solutions have been suggested to resolve this shortcoming. In one approach, HPAs are encapsulated within porous supports. In the second strategy, the supporting material is chemically modified to provide electrostatic interactions with HPA and improve its stabilization on the support. In this line, use of ILs is extensively reported^57^. Moreover, IL not only can act as an anchor to preserve HPA, but also can serve as a catalyst. Therefore, we decorated HR/Y-F127 with IL prior to HPMo stabilization to improve the loading of HPMo and surpass its leaching. To elucidate the role of IL in the catalysis, a control catalyst, HR/Y-F127-HPMo, was prepared through immobilization of HPMo on HR/Y-F127 and its HPMo loading, catalytic activity, DDM selectivity and HPMo leaching (vide infra) were studied and compared with those of HR/Y-F127-IL-HPMo. As summarized in Table 4, the presence of IL in the structure of the catalyst considerably affected the DDM selectivity. More precisely, glycerol acetalization in the presence of HR/Y-F127-IL-HPMo led to 100% conversion and 98% DDM, while in the presence of HR/Y-F127-HPMo, the reaction conversion and DDM yield decreased to 95 and 70%, respectively. Furthermore, the ICP results showed that incorporation of IL in the structure of the catalyst can effectively improve HPMo loading and supress its leaching. This issue can be attributed to the electrostatic interactions between HPMo and IL. Similarly, these interactions can supress the HPMo leaching from HR/Y-F127-IL. These results clearly confirm the role of IL in the catalysis.

**Table 4:** Comparison of the HPMo loading, catalytic activity, DDM selectivity, HPMo leaching of HR/Y-F127-HPMo for glycerol acetalization with those of HR/Y-F127-IL-HPMo.

Catalyst	Conversion (%)	DDM yield (%)	HPMo loading (wt%)	HPMo leaching (% of the initial loading)^a^
HR/Y-F127-HPMo	95	70	0.56	6
HR/Y-F127-IL-HPMo	100	98	0.70	2

^a^Leaching was measured after fifth run of glycerol acetalization via ICP.

### Recyclability of HR/Y-F127-IL-HPMo

Recyclability of catalysts is an essential characteristic for reaching an efficient and feasible catalyst. In this line, the recyclability of HR/Y-F127-IL-HPMo for the acetalization of glycerol was estimated for five consecutive reaction runs. In this regard, glycerol acetalization was conducted under the optimum condition and at the end of the reaction, HR/Y-F127-IL-HPMo was separated and rinsed with distilled water and MeOH for several times to remove the deposited chemicals on the catalyst. Subsequently, the recovered HR/Y-F127-IL-HPMo was dried at 80 °C overnight and applied for the next run of glycerol acetalization. In Figure S4, the reaction conversion and DDM yield of each reaction run are summarized. As illustrated, the conversion of the reaction remained unchanged up to third run of the reaction and slight decrement of this value was observed upon fourth and fifth runs. The effect of reusing of HR/Y-F127-IL-HPMo on the yield of DDM was also negligible and this value decreased from 98 to 91 on fifth run of the reaction.

Low loss of the activity of F127-IL-HPMo can be ascribed to the efficient immobilization of HPMo on HR/Y-F127-IL. To validate this assumption, ICP analysis was utilized to calculate HPMo leaching. Gratifyingly, ICP analysis of the recycled HR/Y-F127-IL-HPMo after fifth run confirmed trivial leaching (2 wt% of the initial loading) of HPMo.

### Plausible mechanism

HR/Y-F127-IL-HPMo is composed of three components, i.e. HR/Y-F127, IL and HPMo. As discussed, the results of the control tests approved that HR/Y-F127 exhibited catalytic activity. On the other hand, the comparative study implied the roles of HPMo and IL in the catalysis. In fact, the utility of HPA for the acetalization of glycerol has been well-established in the literature^58,59^. Considering these results and previous reports^60,61^, it can be suggested that in the course of the reaction, glycerol can be encapsulated in the pores of HR/Y-F127. This issue, known as confinement effect, is beneficiary for the catalysis. More precisely, confinement of the glycerol molecules in HR/Y-F127 pores not only bring them close to the catalytic active sites, HPMo and IL, but also can accelerate the reaction through concentration effect. On the other hand, both IL and HPMo can participate in the catalysis through activation of the carbonyl functionality of acetone. Taking the activity of HR/Y-F127 into account, it can be postulated that HR/Y-F127, that is an H-form zeolite can also take part in the activation of acetone. In the next step, the reaction of the activated acetone with glycerol forms hemiketal as an intermediate that will be dehydrated to generate a tertiary carbonium ion. The latter then tolerates nucleophilic attack of the second hydroxyl group of glycerol to furnish a five-member ring ketal, which then deprotonated to give DDM, Figure S5.

### Comparison of the activity of HR/Y-F127-IL-HPMo with other catalysts

As mentioned before, the importance of glycerol acetalization motivated many researchers to developed catalytic protocols for this chemical transformation. To appraise whether the efficiency of our developed catalytic method is comparable with the previous reports, we compared our results with some of the reported methods that have been carried out under almost similar conditions. As listed in Table 5, HPW catalyst was an efficient catalyst with high DDM selectivity. However, the drawback of this homogeneous catalyst is its unsatisfactory recyclability. Regarding NaY and HR/Y zeolites, the reported results were very similar to our results for our as-prepared control catalysts, i.e. NaY and HR/Y-F127, indicating the low activity and selectivity of microporous NaY and the important role of meso-porosity in achieving high activity and DDM selectivity. Comparison of the performance of Y-W_20_ and HR/Y-W_20_ with their HPA-free counterparts also implied the role of HPA in the catalytic activity. Regarding Nb_15_-HUSY, lower conversion was achieved at higher temperature. This comparison proved that HR/Y-F127-IL-HPMo can be recognized as an efficient catalyst with comparable performance with other catalysts.

**Table 5:** Comparison of the activity of HR/Y-F127-IL-HPMo with other catalysts.

No	Catalyst	G:A^a^	Temp. (ºC)	Time (h)	Conv. (%)	DDM selectivity (%)	DDM yield (%)	References
1	HR/Y-F127-IL-HPMo	1:20	55	2	100	98	98	–
2	HPW	1:20	r.t	2	90	97	87.3	^62^
3	NaY zeolite	1:20	50	2	44.5	22.5	10.0	^15^
4	HR/Y	1:20	50	2	92.7	77.9	72.3	^15^
5	Y-W_20_^b^	1:20	50	2	75.9	68.8	52.2	^15^
6	HR/Y-W_20_^c^	1:20	50	2	98.1	96.4	94.6	^15^
7	Nb_15_-HUSY^d^	1:2	70	3	58	98	57	^63^

^a^Molar ration of glycerol to acetone.

^b^Faujasite zeolite-supported HPW (20 wt%).

^c^Hierarchical faujasite zeolite-supported HPW.

^d^H-form of Ultrastable Y zeolite-supported niobium pentoxide.

## Experimental

### Materials

To synthesize HR/Y-F127-IL-HPMo catalyst, the following reagents and solvents were utilized: phosphomolybdic acid (H_3_[PMo_12_O_40_], HPMo), silicotungstic acid (H_4_[W_12_SiO_40_], HW), phosphotungstic acid (H_3_[PW_12_O_40_], HPW), triethanolamine (TEA), (3-chloropropyl)triethoxysilane (CPTES), sodium hydroxide (NaOH), sodium aluminate (NaAlO_2_), acetic acid, ammonium hydroxide (NH_4_OH), ammonium nitrate (NH_4_NO_3_), PluronicF-127, ludox AS-30 colloidal silica, toluene, ethanol (EtOH), all was provided from Sigma-Aldrich. The used glycerol for the acetalization was purchased from Merck Co., while acetone was provided from Sigma-Aldrich.

### Instruments

Characterization of HR/Y-F127-IL-HPMo catalyst was fulfilled via Fourier transform infrared (FT-IR) spectroscopy using KBr pellet (PERKIN-ELMER‐Spectrum 65 with scan time of 1 s and spectral resolution of 2 cm^−1^), X-ray powder diffraction (XRD) analysis (Inel, EQUINOX 1000 X-ray diffractometer), Scanning electron microscopy (SEM) and elemental mapping analyses (VEGAII TESCAN scanning electron microscope, equipped with QX2, RONTEC energy dispersive X-ray analyzer), thermo gravimetric (TG) analysis (METTLER TOLEDO device, under O_2_ atmosphere within the range of 25–800 °C and heating rate of 10 °C/min). The acidity of the as-prepared samples was evaluated by the temperature programmed desorption of ammonia (NH_3_-TPD(, Chemisorption Analyzer, NanoSORD (made by Sensiran Co., Iran), the heating ramp rate was 20 °C/min, in the temperature range of 25–800 °C). To measure the specific surface area (S_BET_) and total pore volume (V_P_) of the catalyst, Brunauer–Emmett–Teller (BET) method (BELSORP MINI II, BEL apparatus) was applied. The catalyst pre-heating step was done at 150 °C for 3 h. Average pore diameter (D_P_), micropore volume (V_micro_) and mesopore volume (V_meso_) were obtained via BJH and t-plot methods.

To identify the reaction products and calculate their yields, GC analysis (Shimadzu GC 17A apparatus, equipped with flame ionization detector (FID) and fitted with a Carbowax capillary column) was employed. In this analysis, the temperature of detector and injector were fixed on 280 and 250 °C, respectively.

### Synthesis of the catalyst

#### Synthesis of hierarchical faujasite zeolite: HR/Y-F127

In this project, for the first time a hierarchical faujasite zeolite, HR/Y-F127, was prepared through post synthesis (Top-down) approach using PluronicF-127 as surfactant. The synthetic procedure consists of several steps. In the first step, NaY zeolite was synthesized. For this purpose, sodium aluminate (13.5 g) and NaOH (10 g) were dissolved in deionized water. Then, silica sol (100 g, ludox colloidal silica, 30 wt%) was added and the resulted mixture was stirred for 4 h. Subsequently, the mixture was aged at ambient temperature for 3 days and then hydrothermally treated in a Teflon lined autoclave at 95 °C for 3 days. Upon completion of the reaction, the reactor was cooled and the precipitate was filtered and repeatedly washed with deionized water to reach pH < 10. After that, the solid was dried at 110 °C for 12 h.

In the next step, the as-prepared NaY was dealuminated. In this regard, NaY (2 g) was mixed with acetic acid (0.03 N, 60 mL) and stirred for 2 h at 50 °C. Afterwards, the solid was filtered, rinsed with deionized water and dried in oven at 110 °C for 12 h. Next, PluronicF-127 was used for soft templating of the delaminated NaY. Briefly, to the suspension of dealuminated NaY (1 g) in NH_4_OH solution (0.3 M), PluronicF-127 (0.3 g) was added and the resulting mixture was stirred at 40 °C for 1 h and then transferred to an autoclave and hydrothermally treated at 150 °C for 16 h. Upon completion of the reaction, the precipitate was filtered, rinsed with deionized water and dried at 80 °C overnight. In the next step, the obtained zeolite was treated with NH_4_NO_3_ (1 N) at 80 °C for 2 h to furnish the H-form zeolite. Finally, the removal of the template was achieved via calcination at 550 °C for 5 h. The obtained product was then steamed at 600 °C for 4 h to give ultra-stable hierarchical zeolite, denoted as HR/Y-F127.

#### Functionalization of HR/Y-F127 with IL: synthesis of HR/Y-F127-IL

To functionalize HR/Y-F127 with IL, a two-step procedure was utilized. First, HR/Y-F127 (2 g) was suspended in dry toluene and stirred for 20 min. Afterwards, CPTES (2 mL) was slowly introduced into the reaction vessel and the obtained mixture was refluxed at 110 °C under Ar atmosphere for 24 h. At the end of the reaction, the precipitate, HR/Y-F127-Cl, was separated, washed repeatedly with dry toluene and dried at 80 °C overnight. In the next step, HR/Y-F127-Cl was reacted with TEA to furnish HR/Y-F127-IL. In more detail, HR/Y-F127-Cl (2 g) was suspended in EtOH (40 mL) and stirred for 15 min. Subsequently, TEA (5 mmol) was added and the reaction mixture was agitated for 24 h under reflux condition. Upon completion of the reaction, the product was separated via centrifugation and rinsed with EtOH several times. Finally, the as-prepared support, HR/Y-F127-IL, was dried in oven at 80 ºC overnight.

#### Impregnation of HPMo: synthesis of HR/Y-F127-IL-HPMo

Wet-impregnation method was utilized for the immobilization of HPMo on HR/Y-F127-IL. Typically, HPMo aqueous solution (20 wt%) was gently added to the stirring suspension of HR/Y-F127-IL (2 g) in deionized water. Mixing was continued for 24 h at room temperature. Then, the solid, HR/Y-F127-IL-HPMo, was separated via centrifugation, washed with deionized water and dried in oven at 80 °C overnight. Using ICP analysis, the loading of HPMo was measured as 0.7 wt%.

### Acetalization of glycerol

To evaluate the catalytic performance of HR/Y-F127-IL-HPMo in glycerol acetalization, a mixture of glycerol (1 mol) and acetone (20 mol) was prepared and heated at 55 °C. Then, the proper dosage of HR/Y-F127-IL-HPMo catalyst (5–10 wt%) was added to the mixture and the reaction was continued under reflux condition for 2 h. At the end of the reaction, HR/Y-F127-IL-HPMo was removed from the reaction mixture via centrifugation and the reaction products and their yields were determined via GC analysis. The following equation (Eq. 1) was applied to measure the reaction conversion^15^:

1 $${\text{Conversion }}\left( \% \right) = \left( {{\text{M}}_{{{\text{GF}}}} - {\text{M}}_{{{\text{GO}}}} } \right)/{\text{M}}_{{{\text{GF}}}} \times 100$$

where M_GF_ and M_GO_ were moles of glycerol in the feed and output, respectively.

The selectivity of the reaction towards DDM and DDM yield were calculated via Eqs. 2 and 3, respectively.

2 $${\text{Selectivity }}\left( \% \right) = {\text{ M}}_{{\text{S}}} /\left( {{\text{M}}_{{{\text{GF}}}} - {\text{ M}}_{{{\text{GO}}}} } \right) \, \times { 1}00$$

where Ms accounts for mol of DDM.

3 $${\text{Yield }}\left( \% \right) = \left( {{\text{Conversion }}\left( \% \right) \, \times {\text{ Selectivity }}\left( \% \right)} \right)/{1}00$$

## Conclusion

To introduce mesoporosity in NaY zeolite, a novel approach has been devised, in which NaY was first partially dealuminated through acid-treatment and then treated with PluronicF-127 template hydrothermally. The resultant compound was subsequently reacted with NH_4_NO_3_ to furnish the H-form zeolite and then calcined to remove the template. BET analysis approved formation of mesopores in the structure of the catalyst. In the next step, the as-prepared hierarchical zeolite, HR/Y-F127, was functionalized with IL and used as a support for the immobilization of HPA to furnish HR/Y-F127-IL-HPMo. The catalyst was fully characterized and then applied for promoting glycerol acetalization under solvent-less condition. The effects of the reaction variables were investigated. Moreover, the effects of mesoporosity of the zeolite and incorporation of IL on the high conversion and DDM yield were approved by comparison of the catalytic performance of NaY and HR/Y-F127. Furthermore, the role of the nature of Keggin type HPA was appraised by using three different Keggin type HPAs. The recyclability tests also affirmed the recyclability of the catalyst as well as low HPA leaching.

### Supplementary Information

Supplementary Information.

## Data Availability

All data used and/or analyzed during the current study are presented in the article. To access the data, all can contact Sara Tarighi (s.tarighi@ippi.ac.ir).
