# Supplementary material for: Heteropolyacid supported on ionic liquid decorated hierarchical faujasite zeolite as an efficient catalyst for glycerol acetalization to solketal
Source: Sci Rep. 2023 Sep 21;13:15703. doi: 10.1038/s41598-023-42956-8 (PMC10514292; doi:10.1038/s41598-023-42956-8)
Supplement: Supplementary file 1 — Supplementary Information. [file 41598_2023_42956_MOESM1_ESM.docx]

**Supplementary information**

**Heteropolyacid supported on on ionic liquid decorated hierarchical faujasite zeolite as an efficient catalyst for glycerol acetalization to solketal**

Samahe Sadjadi*^a1^, Sara Tarighi*^1^, Motahareh Delangiz^a1^, Majid Heravi*^2^

**
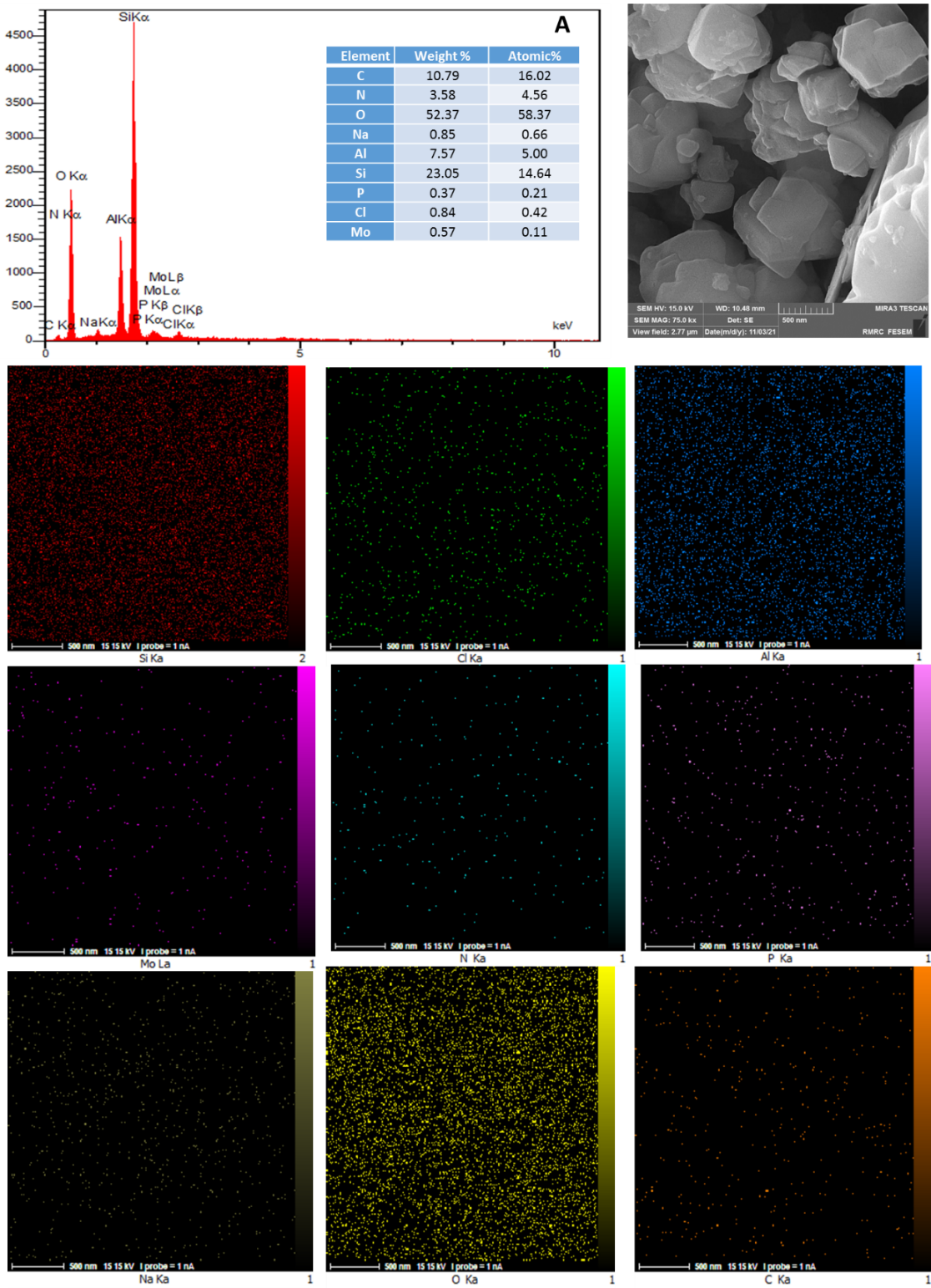
**

**B**

**Figure S1.** A: EDS and B: elemental mapping analyses of the catalyst.

**Figure S2.** NH_3_-TPD profiles of HR/Y-F127 and HR/Y-F127-IL-HPMo

**Figure S3.** N_2_-adsorption-desorption isotherms of HR/Y-F127 and HR/Y-F127-IL-HPMo

**Figure S4.** The results of recyclability of HR/Y-F127-IL-HPMo for acetalization of glycerol under optimum condition.

**Figure S5.** The proposed mechanism for glycerol acetalization to DDM.
